# The Burden of Serious Fungal Infections in Tajikistan

**DOI:** 10.3390/jof5030068

**Published:** 2019-07-21

**Authors:** Oktam I. Bobokhojaev, Ali Osmanov, Samariddin P. Aliev, Asliddin S. Radjabzoda, Ziyovuddin T. Avgonov, Safarbek T. Manonov, David W. Denning

**Affiliations:** 1 Department of Phthisiopneumology, Tajik State Medical University, 734003 Dushanbe, Tajikistan; 2 Global Action Fund for Fungal Infections, 1208 Geneva, Switzerland; 3 Research Scientific Institute of Preventive Medicine, Ministry of Health and Social Protection of the population, 734025 Dushanbe, Tajikistan; 4 Republican Center of the Protection Population from Tuberculosis Ministry of Health and Social Protection of the Population, 734000 Dushanbe, Tajikistan; 5 Secretariat of the National Coordination Committee to Fight AIDS, TB and Malaria, 734018 Dushanbe, Tajikistan; 6 Republican Center of Medical Statistics, Ministry of Health and Social Protection of the Population, 734025 Dushanbe, Tajikistan; 7 Faculty of Biology, Medicine and Health, University of Manchester, Manchester Academic Health Science Centre, Manchester M13 9NT, UK; 8 National Aspergillosis Centre, Wythenshawe Hospital, Manchester University NHS Foundation Trust, Manchester Academic Health Science Centre, Southmoor Road, Manchester M23 9LT, UK

**Keywords:** Tajikistan, fungal infection, aspergillosis, candidiasis, epidemiology

## Abstract

Tajikistan is a low-income country in Middle Asia with a population of 8.9 million people. Five percent of the population lives on less than 1.9 USD a day and 54% live on less than 5.5 USD a day. We have estimated the burden of serious fungal infections in Tajikistan. It was estimated that 168,834 Tajik women develop recurrent vulvovaginal candidiasis. Among HIV-positive patients, we estimate 490 patients with oesophageal candidiasis and 1260 patients with oral candidiasis, 41 cases of cryptococcal meningitis and 210 cases of *Pneumocystis* pneumonia annually. According to our estimations there are 774 cases of chronic pulmonary aspergillosis (CPA) as a sequel of tuberculosis; CPA may occur as a consequence of multiple pulmonary conditions and the total prevalence of 4161 cases was estimated. We have estimated 6008 cased of allergic bronchopulmonary aspergillosis (ABPA) and 7930 cases of severe asthma with fungal sensitisation (SAFS), and 137 fungal asthma deaths annually. We have estimated 445 cases of candidemia a year applying a low European rate. There are approximately 283 cases of invasive aspergillosis annually. There are 189,662 (2.1% of the population) people suffering from serious fungal infections in Tajikistan. Hence, improving diagnostics is the first step of understanding a scale of the fungal burden.

## 1. Introduction

The burden of invasive fungal infections increases worldwide due to the various factors that include increased survival from previously fatal illnesses and increasing numbers of immunocompromised patients [1]. More than 150 million people have serious fungal infections worldwide while the mortality associated with fungal infections is similar to tuberculosis and more than 3 times higher than the mortality from malaria [2]. Still, fungal infections may be considered as a ‘neglected epidemic’ being one of the major causes of mortality among immunocompromised patients [3], with fatality ratios up to 70% and almost 100% if untreated [4].

Understanding of the burden of serious fungal infections is limited due to the insufficient diagnostic capabilities and lack of awareness of this problem. An important initial step is to quantify the burden of these infections and highlight the problem on national and international levels. Since 2013, the burden of serious fungal infections was estimated for more than 80 countries in collaboration with the Leading International Fungal Education (LIFE). There were no previous attempts for such an estimation in Tajikistan and this work aims to cover this gap. 

**Figure 1:**
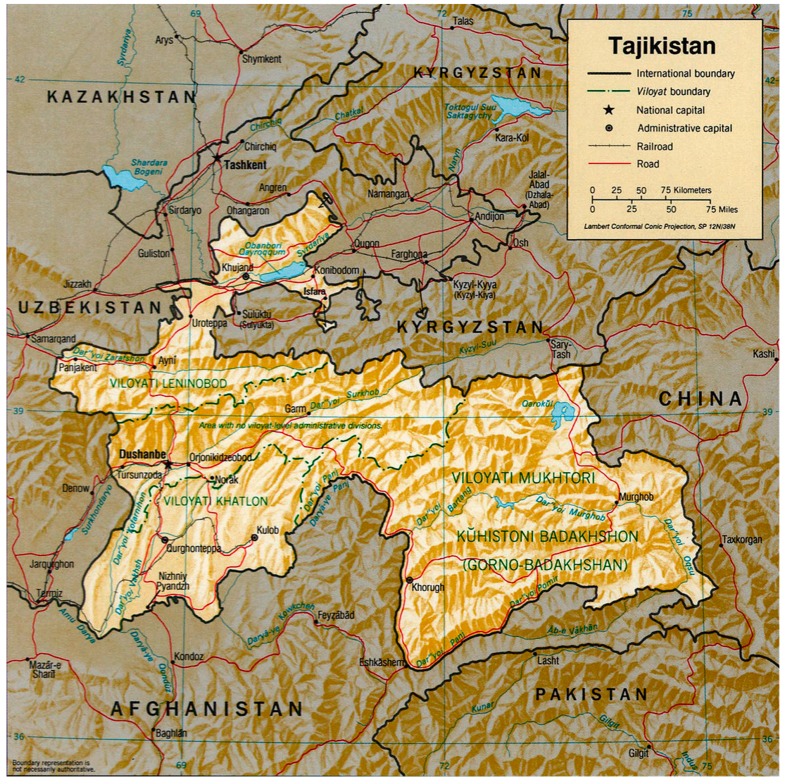
Geography of Tajikistan [7].

The Republic of Tajikistan in Central Asia with a population of approximately 8.9 million people (Figure 1). There are more men (52%) than women (48%) and more than 70% of the population lives in rural regions (Ministry of Health and Social protection, Tajikistan). Tajikistan is one of several ‘young’ countries with the average age of the population being 24.0 years. The natural population increase is 24.2 per 100,000 population. 

Tajikistan is a low-income country with a projected GPD per capita in 2019 of 1054 USD [4]. The poverty rates are high with the number of people living below $2 purchasing power parity (PPP) per day is 4.8% of the population (427,000 people) and the number of people living below $5.5 PPP per day is 52% (4.6 M people) of the population [5]. Malnutrition is one of the main healthcare problems in Tajikistan [6].

## 2. Materials and Results

The previously published LIFE model was used to estimate the burden of serious fungal infections in Tajikistan [8]. The first step was to identify all published papers on the burden of each serious fungal infection in English, Tajik, and Russian languages via several databases, namely ‘cyberleninka’, ‘elibrary.ru’, ‘Google Scholar’, and ‘PubMed’. The search period includes all dates up to June 2019. Unfortunately, there are no published data on this topic. So, we have used deterministic modelling developed by LIFE to make estimates based on each population ‘at-risk’. The total burden of serious fungal infections and the rate per 100,000 population are summarized in Table 1.

There are more than 14,000 people living with HIV (PLHIV) in Tajikistan, and 70% of them do not receive antiretroviral therapy (ART) [9]. In 20% of patients with CD4 cells < 200 per mL and in 5% of patients receiving antiretroviral therapy, oesophageal candidiasis occurs at least once, while oral candidiasis occurs in 90% of patients with CD4 cells < 200 per mL [10,11,12]. We assumed a 7-year decline in the number of CD4 cells in patients not receiving ART, so we estimated 490 patients with oesophageal candidiasis and 1260 patients with oral candidiasis annually. We have assumed a prevalence of cryptococcal antigenemia of 2.9% [13], which results in approximately 41 cases of cryptococcal meningitis. *Pneumocystis* pneumonia (PCP) occurs in 15% of patients presenting with AIDS [14], hence we estimated 210 cases of PCP annually. Probably other cases of PCP occur in other immunocompromised patients, but we were not able to estimate them.

According to WHO there were 6279 cases of tuberculosis (TB) in 2017, of which 4883 cases were pulmonary TB [15]. Chronic pulmonary aspergillosis (CPA) complicates 13–23% of pulmonary TB [16,17,18], and assuming 15% annual mortality and 6% resection rate, we estimated 1040 cases of CPA as a sequel of TB [19]. Some patients with CPA do not have a history of TB and present in a similar way to TB. They are smear and GeneXpert negative. In addition, CPA occurs as a sequel of other conditions such as emphysema, sarcoidosis and pneumothorax. According to Smith et al. [20], the prevalence of CPA in these conditions is three times higher than in the prevalence of post-TB CPA. Hence, there are approximately 4161 cases of CPA in total in Tajikistan, remarkably high rate of 46.8/100,000 [20]. 

The prevalence of asthma in Tajikistan is not known, but To et al. [21] estimates between 2.77% to 2.9% of adults suffer from asthma. These estimates provide us with 240,320 of asthma patients in Tajikistan. As allergic bronchopulmonary aspergillosis (ABPA) occurs in approximately 2.5% [22] of asthma patients, there are approximately 6008 patients with ABPA in Tajikistan. There are no data on fungal sensitisation (measured by IgE levels) in patients with asthma in Tajikistan, to assess if 2.5% is the correct proportion of patients with ABPA. However, this same figure was found in Saudi Arabia and in China [23,24]. Using 33% sensitization rate in the most poorly controlled group (10%), we have estimated 7930 patients with severe asthma with fungal sensitisation (SAFS) [25]. There are 196 annual deaths from asthma, which gives us 137 fungal asthma deaths annually due to the fact that 70% of people repeatedly admitted to hospital with asthma have fungal sensitization [26]. 

There are no local data on chronic obstructive pulmonary disease (COPD) prevalence; however, according to Adeloye et al. [27], COPD prevalence in this region is 13.20% of the over-30 age group. Hence, there are probably 393,680 patients with COPD in Tajikistan. According to Polati et al [28], approximately 5% of patients with COPD are hospitalized at least once a year. As invasive aspergillosis (IA) occurs in 1.3% of hospitalized COPD patients [29], we have approximated 256 cases of IA in these patients. However, in Southern China the IA incidence in hospitalized patients was 3.9% [30], which means that our estimates of IA in COPD patients are conservative. The five-year prevalence of patients with cancer was 9987, while there are 332 patients with lung cancer [31]. We have assumed the rate of IA being 2.6% in lung cancer patients [32], which provides us nine cases in this group of patients. The rate of invasive aspergillosis is found in at least 10% of patients with AML not given effective antifungal prophylaxis and all other forms of leukaemia [33]. Assuming AML rate of 4.7/100,000 [34], there are eight cases of IA in patients with leukaemia. There were 114 solid organ transplants in 2018, out of them 104 renal transplants and 10 partial liver transplants. These mean that there was only one case of invasive aspergillosis in these groups [35]. As a result, the total number of patients with invasive aspergillosis in Tajikistan is estimated to be 274.

Recurrent vulvovaginal candidiasis (rVVC) is defined as having four or more episodes per year. There are 2,813,903 women between 15 and 50 years who are at-risk for developing rVVC; assuming the rate of 6%, we have estimated that 168,834 Tajik women suffer from rVVC [36,37]. The number of surgical beds was obtained from the Ministry of Health and comprised 26,892 per annum [38]. We used a low-European average of 5.0 per 100,000 to estimate 445 patients with candidemia [39]. Approximately 30% of candidemia cases occur in ICUs, which means that there are approximately 148 cases of candidemia occurring in ICUs in Tajikistan [40]. There are no data on candida peritonitis complicating peritoneal dialysis. French data show that, for every two patients in with candidemia in intensive care, there is one case of postsurgical candida peritonitis/intra-abdominal candidiasis which implies 74 intra-abdominal candidiasis cases [39].

We found no data on histoplasmosis, mucormycosis, mycetoma, chromoblastomycosis, or fungal keratitis. 

## 3. Discussion

There are probably 189,662 (2.1% of the population) people who are suffering from serious fungal infections in Tajikistan. Amongst them, 979 people have imminently life-threatening conditions (*Candida* peritonitis, candidemia, cryptococcosis, *Pneumocystis* pneumonia, and invasive aspergillosis). In Tajikistan, the main drivers of serious fungal infections are respiratory diseases, particularly TB and COPD, while the number of immunocompromised patients is relatively small. 

The structure of fungal burden in Tajikistan is different to Ukrainian, Russian, and Uzbek burdens [8,41,42]. This is caused by differences in the prevalence of underlying conditions. Tajikistan has high rates of chronic pulmonary aspergillosis (46.8/100,000) driven by a high burden of TB and respiratory diseases. In comparison, CPA rates in Ukraine and Uzbekistan are 22 and 6.3 per 100,000 population correspondingly. On the other hand, in Russia the rate of CPA is very high (126.1/100,000) due to the epidemic of poorly controlled pulmonary TB.

At the same time, the rates of cryptococcal meningitis, *Pneumocystis* pneumonia, oral and oesophageal candidiasis are low in Tajikistan due to the low numbers of PLHIV. The healthcare system of Tajikistan is low-resourced and, as a result, the number of immunocompromised patients is low and very few undergo solid organ transplants [43]. This results in low numbers of IA among immunocompromised patients while the putative number of IA cases among patients in ICU remains high.

In addition to the fungal burden, there are several factors that increase urgency of the problem. First, the only available diagnostics methods are culture-based. Second, itraconazole, voriconazole, caspofungins, flucytosine, and liposomal forms of amphotericin B are not available in Tajikistan. The only available antifungals are conventional amphotericin B and fluconazole [44]. Third, there are no national educational programmes on diagnostics and treatment of fungal infections, and no critical mass of mycologists (clinical, laboratory or research). 

Emerging of antifungal resistance is a growing threat to public health because it drastically decreases treatment options and increases morbidity and mortality, duration of hospitalization, and healthcare costs [45]. *Candida auris* and azole-resistant strains of *Aspergillus* spp. are the most challenging for medical community. *C. auris* has caused nosocomial outbreaks on five continents and bears inherent drug-resistance [46]. Azole resistance of *Aspergillus* species is driven by clinically used antifungals but also by agricultural use of azole agents [47]. Although, there is a multinational initiative on systemic antimycotic and antifungal use [48], there is not much data on Tajikistan since only available systemic antifungals are fluconazole and conventional amphotericin B. 

Although superficial fungal infections are one of the most common infections, affecting approximately 1.7 billion people [49], we were not able to estimate the burden of superficial fungal infection due to absence of national surveillance programs and due to the fact that there is no population ‘at-risk’ for these infections. 

Tajikistan soil is a natural habitat for a pathogenic fungus *Scedosporium apiospermum* (formerly *Pseudallescheria boydii* and *Allescheria boydii*) [50,51]. This therapy-refractory fungus causes disseminated infections in immunocompromised patients, namely endophthalmitis, meningitis (often after near drowning), osteomyelitis, pneumonitis, and prosthetic valve endocarditis [52]. The clinical significance of *S. apiospermum* in cystic fibrosis patients is high as it colonizes lungs [53]. However, there is also evidence of this fungus infecting immunocompetent individuals [54]. The burden of these infections in Tajikistan is not known.

This estimation is the first step of highlighting this problem to national and global healthcare authorities. The next essential steps should be performed simultaneously namely: i) creating education programmes for healthcare professionals; ii) providing availability of diagnostics methods; iii) provision of essential antifungals. 

## Figures and Tables

**Table 1:** The burden of serious fungal infections in Tajikistan.

Infection	Number of Infections Per Underlying Disorder Per Year					Rate/100K	Total Burden
	None	HIV/AIDS	Respiratory	Cancer/Tx	ICU		
Oesophageal candidiasis	-	490	-	-	-	5.5	490
Oral candidiasis	-	1260	-	-	-	14.1	1260
Candidemia	-	-	-	-	371	4.2	371
Candida peritonitis	-	-	-	-	74	0.8	74
Recurrent vaginal candidiasis (4x/year+)	168,834	-	-	-	-	3794	168,834
Allergic bronchopulmonary aspergillosis	-	-	6008	-	-	67.5	6008
Severe asthma with fungal sensitisation	-	-	7930	-	-	89.1	7930
Fungal asthma deaths	-	-	137	-	-	1.5	137
Chronic pulmonary aspergillosis	-	-	4161	-	-	46.8	4161
Invasive aspergillosis	-	-	-	27	256	3.2	283
Cryptococcal meningitis	-	41	-	-	-	0.5	41
*Pneumocystis* pneumonia	-	210	-	-	-	2.4	210
Fungal keratitis	?	-	-	-	-	?	?
Tinea capitis	?	-	-	-	-	?	?
Total burden estimated	168,834	2001	18,099	27	701		189,662

‘-’ None; ‘?’ not known.
